# Tumors of the Face

**Published:** 1835

**Authors:** Horatio G. Jameson

**Affiliations:** Professor of Surgery in the Cincinnati College


### The Western Journal

OF THE

### MEDICAL AND PHYSICAL SCIENCES.

FOR JULY, AUGUST, AND SEPTEMBER, 1835.

### ESSAYS AND CASES.

### Art. I.—Tumors of the Face.

*Notices of two cases of Tumors of the Face, in which operations were performed.* By Horatio G. Jameson, M. D. Professor of Surgery in the Cincinnati College.

Case First.—Mr. C——, from Virginia, visited Baltimore in the month of February, 1835, with the intention of putting himself under my care, in a case of tumor of considerable magnitude. I found a tumor equal in size to a moderate sized lemon, extending from the zygomatic process of the left cheek, with its longest diameter downwards, and lying close along the ear. Neither the sight nor touch discovered any derangement of the bones internally; below the teeth of the upper jaw, there was a slight projection inward, of a small lobe of the tumor, covered by the lining of the mouth. The case had been attended from an early period with severe neuralgic pain, which might be ascribed to the pressure of the tumor on the portio dura of the seventh nerve, near its escape from the skull. Health of the patient good, except a considerable degree of debility, arising from long continued and severe pain, such as to require the free use of opium. By grasping the tumor firmly with the thumb and fore-finger, there was evidently a slight degree of motion discovered, in the lower part of the tumor.

In forming an opinion, so far as it could be done by the external examination, I was led to hope, that the bones of the face were not involved in the disease; but the operation disclosed considerable absorption of the bone and muscle.

*The Operation.*—An incision was made commencing over the zygomatic process, and carried downwards and outwards to a point a little below the cartilagenous tube of the ear, about three inches in extent; another was now started at this point, and carried about two inches towards the corner of the mouth—the integument being dissected up over the greater part of the tumor, I now commenced at the upper portion of the tumor to dissect it out. I soon found that the tumor descended deep into the cheek, and that considerable absorption of the muscles, and a portion of the outer plate of the superior jaw bone, had taken place. Instead of finding the tumor in any part encysted, as I had hoped, it was attached to all the adjacent parts; and, when I had penetrated below the attachments to the soft parts, I found the tumor in actual contact with the bony surface. A branch of the tumor had penetrated the outer portion of the antrum highmoriamum—all of the bony surface looked healthy. The tumor readily peeled off from the bone. The maxilliary bone presented a very sharp and ragged end, where it had been resting closely against the tumor; this projection I sawed off with Hey's saw, and left a smooth, sound surface. The parotid gland, being closely attached to the tumor, was in greater part removed—several arteries were cut, and tied, or treated by tortion. Having satisfied myself, and friends present, that the diseased parts were removed, I now examined the parts with a view to dressing the wound.

When I looked at the flaps of skin, presenting an area of several inches, having nothing but the vessels of the skin, to sustain the covering for the wound, I was truly alarmed, lest the extent of detached integument was too great to allow of a reunion of the divided parts. When the flaps on either side were turned down, and made to meet, it was seen that there would be a vast cavity between the outer covering and the bottom of the wound; these flaps passing from the upper side of the wound on either edge, and crossing over the cavity below, made by the absorption already noticed, presented a state of things requiring the most minute attention, in the coaptation of the *flaps*. Much difficulty arose from the floating or loose state of the skin, owing to its tendency to fall inward into the cavity, or descend upon the slightest pressure.

Three interrupted sutures of fine doubled thread were introduced with much care, then was commenced at the upper angle, the insertion of very fine cambric needles, at intervals of less than the fourth of an inch: and, having brought these down to the first suture of thread, the needles were now lapped in the usual manner, with fine thread, taking the utmost care to keep the edges of the cut skin precisely on a level. This, though an affair very simple to an ordinary apprehension, is really one of the most delicate operations in surgery. In the present case, the floating condition of the flaps, and extensive hollow below, rendered this unquestionably that part of the operation requiring most skill. Skill here consists in knowing how to lift or hold up the loose edges, while we at the same time pass the needle through, and so regulate it as to obtain an exact fitting between the separated edges. The introduction and lapping of the needles being completed, leaving a little space open on the lower incision, for the escape of matter, the part was dressed by applying a soft piece of patent lint, and confining it with a roller.

To my great gratification, I found on inspection on the second day, that complete adhesion had taken place throughout the whole wound, so as to require the introduction of a probe, where provision had been made for the exit of matter. A considerable discharge continued for several days, of a thin, bloody fluid—soon, however, pus began to appear, and in about ten days there was a pretty free discharge of laudable pus. The patient continued to suffer neuralgic pains of the forehead, on the affected side, but they were easily controlled by the use of opium, and gradually his condition in all respects became more agreeable and healthful.

Two weeks after the operation, I left Baltimore, for the south, and put the patient in the care of my friend, Dr. Annan, doing well, as I believed. He left town a few days afterwards, since which time I have not heard from him.

An examination of the tumor and the improving condition of my patient, led me to hope that he would recover; but still the operation was too long delayed, and considerable deformity unavoidably followed the cure, from the deep and extensive depression left in the cheek.

The tumor was in greater part steatomatous, but traces were not wanting of the arborescent structure, so often seen in malignant tumors.

I deem this case interesting from the fact of having succeeded so well, in the reunion of so vastly great a portion of integument, without having any living structure in contact with all its inner surface.

During the swelled state of the parts the skin was tightly stretched across the cavity; but, as the healing progressed, the skin seemed to adhere to the surface below, at the edges; and gradually extended inward, so as to present a most pleasing effort of nature to effect a cure—the skin became nicely tucked in around the edge of the projecting bone, and settled down gradually to the bony surface. The approximation and union of surfaces was promoted by suitable compression fitted to the hollow.

Case Second.—I was called into Pennsylvania, by my friend, Dr. Benjamin Johnston, in the month of July, 1835, for the purpose of operating upon Mr. Strickler, residing near York. The patient is about 70 years of age, of correct habits, and firm constitution; in good health, possessed of uncommon fortitude. He has a tumor on the right side of his face, equal in size to a large orange; it occupies a large portion of the cheek, extending from the zygoma to a little under the angle of the jaw; reaching nearly from the corner of the mouth to the ear, and is closely attached to the tube of the external ear. It is obviously moveable, and the fingers can be passed under the point of it, which creeps somewhat below the angle of the inferior jaw. There has not been much pain, and the skin over the tumor is still loose, and easily moves over the surface of the tumor, when the fingers are applied for that purpose. The thickest part of the tumor lies on the parotid gland, and we may presume, that if there be not absorption of the gland, that it is materially deranged in its function, and probably deranged in its structure. At all events, it is highly probable, that such are the attachments to the gland, and the depth of the tumor, that the latter can only be removed by the destruction of the former. In this event, the *portio dura* may be destroyed, and paralysis follow, of this side of the face.

The patient stated, with much good sense, that he had sent for me to obtain my counsel, that without regard to present suffering, he was ready to submit his case to my judgment, feeling assured that I would not operate unless it could be done with propriety.

Having carefully examined the tumor, in all its attachments, its history and character, I did not hesitate to advise its removal, because, whatever might be its course hereafter, at present it exhibited nothing strongly indicative of malignancy; and as there was every reason to hope that it could yet be entirely removed, I did not hesitate to advise an operation, seeing that ere long it would not only be beyond our reach, but death must shortly take place, since the tumor is now growing with rapidity, and principally downwards and under the angle of the lower jaw. Doctors Johnston and Jones, who were present, concurred in the opinion, that the removal was proper, as well on account of the nature of the disease, as of its practicability, though certainly a very painful, difficult, and extensive operation. Our patient had the necessary stamina, whether we allude to his corporeal or mental powers.

The patient having been made acquainted with the above view of his case, submitted manfully, and without a moment's hesitation proceeded to prepare for the operation.

The base of the tumor was irregularly circular, except a small projection downwards under the angle of the jaw.

An incision was made from the zygoma to the angle of the jaw, near the posterior margin of the tumor; another from the anterior edge of the divided skin, about two and a half inches towards the mouth. I soon discovered the tumor to be closely attached to everything with which it lay in contact, and that it required to be dissected throughout its entire surface; and, being in part steatomatous and irregular, rendered the operation tedious and painful. Being uncertain as to the condition of the parotid, and of the parotid duct, I proceeded with much caution, that I might avoid, if possible, doing injury to these structures; but, such was the depth of the tumor, so irregular its inner surface, such the confusion of parts, from pressure, induration, universal and copious hemorrhage; that I found it necessary to proceed sans ceremonie, to take out the diseased mass. The separation of the attachment of the tumor to the cartilaginous tube of the ear, and under the edge or angle of the jaw, required great care. The tumor was abundantly supplied with blood, all the smaller arteries being greatly enlarged. Some of these it was necessary to tie as we proceeded, because they were so situated that a finger could not be applied, without incommoding the operator; others were restrained by the fingers of my assistants, until the tumor was extirpated. Not less than eight or ten arteries required ligatures, and I was sensibly struck with the fact, that however well we may succeed generally, by tortion in securing arteries of the size here wounded, in this case such seemed to be the force of the circulation, that it would not restrain the hemorrhage, and it became necessary to tie every artery.

Upon examining the wound after the removal of the tumor, I discovered that there had been a very intimate union of the tumor to the parotid gland. The long continued pressure, of many years' duration, had much wasted the gland; and it is most likely that little or none of its natural function was now operative, and that the duct had been impervious. Nearly all of the gland had been removed, but since no paralysis took place, the portio dura could not have been destroyed. The carotid artery lay beating violently upon the surface of the wound, and a degree of pulsatory movement existed, at so many points as to present rather an appalling spectacle, to see all alive with leaping arteries.

The operation, though tedious and very painful, was submitted to without a murmur, on the part of the patient; and when told it was done, he remarked that he had not expected to get so soon through the operation. Much pains were taken to satisfy ourselves by sponging and removing coagula, that all the vessels were secured. The wound was then laid neatly together, and the edges secured by a few interrupted thread sutures; then the interspace was most carefully closed by means of fine cambric needles, which were lapped in the usual manner with fine thread.

We had the gratification to see nearly all of this extensive wound healed by the first intention. There was neither palsy of the face, nor fistulous disposition; on the contrary, the parts healed kindly, with but little deformity, and in three weeks the whole wound was completely healed.

We would beseech our brethren abroad, both in this country and throughout Europe, to look at the result of this case, wherein eight or ten ligatures were applied, and union by the first intention not interrupted—and yet we are told every day, of ligatures coming away many days after application. Does it matter not whether patients be subjected to the delay, irritation, pain, inflammation, and suppuration, which always attend indissoluble ligatures? Can the profession not be brought to perceive the difference between suffering or no suffering? between safety and danger?—for most assuredly these differences exist between the animal and hard ligatures. I know of nothing in the practice of surgery so consoling, so truly gratifying, as the happy consequences attendant upon the animal ligature.

